# Continuous venovenous hemodiafiltration versus standard medical therapy for the prevention of rhabdomyolysis-induced acute kidney injury: a retrospective cohort study

**DOI:** 10.1186/s12882-023-03242-x

**Published:** 2023-07-19

**Authors:** Yan Meng, Ming-xue Zhou, Chun-bo Wu, De-hua Wang, Jian-rong Zhao, Dong-yin Shi

**Affiliations:** 1 Department of Nephrology, The Affiliated Hospital of Inner Mongolia Medical University, Hohhot, 010050 Inner Mongolia PR China; 2 Department of Nephrology, Chifeng Municipal Hospital, Chifeng, China; 3 Department of Interventional Medicine, the Fifth Hospital of Shijiazhuang, Shijiazhuang, China

**Keywords:** Continuous renal replacement therapy (CRRT), Continuous venovenous hemodiafiltration (CVVHDF), Rhabdomyolysis, Acute kidney injury, Health economics

## Abstract

**Aim:**

To determine whether continuous venovenous hemodiafiltration (CVVHDF) plus standard medical therapy (SMT) vs. SMT alone prevents rhabdomyolysis (RM)-induced acute kidney injury (AKI) and analyze the related health economics.

**Methods:**

This retrospective cohort study involved 9 RM patients without AKI, coronary heart disease, or chronic kidney disease treated with CVVHDF plus SMT (CVVHDF + SMT group). Nine matched RM patients without AKI treated with SMT only served as controls (SMT group). Baseline characteristics, biochemical indexes, renal survival data, and health economic data were compared between groups. In the CVVHDF + SMT group, biochemical data were compared at different time points.

**Results:**

At 2 and 7 days after admission, serum biochemical indices (e.g., myoglobin, creatine kinase, creatinine, and blood urea nitrogen) did not differ between the groups. Total (*P* = 0.011) and daily hospitalization costs (*P* = 0.002) were higher in the CVVHDF + SMT group than in the SMT group. After 53 months of follow-up, no patient developed increased serum creatinine, except for 1 CVVHDF + SMT-group patient who died of acute myocardial infarction. In the CVVHDF + SMT group, myoglobin levels significantly differed before and after the first CVVHDF treatment (*P* = 0.008), and serum myoglobin, serum creatinine, and blood urea nitrogen decreased significantly at different time points after CVVHDF.

**Conclusions:**

Although CVVHDF facilitated myoglobin elimination, its addition to SMT did not improve biochemical indices like serum myoglobin, serum creatine kinase, creatinine, blood urea nitrogen, and lactate dehydrogenase or the long-term renal prognosis. Despite similar hospitalization durations, both total and daily hospitalization costs were higher in the CVVHDF + SMT group.

## Introduction

Rhabdomyolysis (RM) is a clinical syndrome characterized by the injury and disintegration of skeletal muscle fibers followed by the release of myoglobin, electrolytes, enzymes, and other components of the injured skeletal muscle cells into the blood circulation [1, 2]. RM can be caused by a variety of traumatic and non-traumatic factors, such as excessive exercise, crush injuries, statins, poisoning, infections, and autoimmune diseases. The main clinical manifestations of RM are muscle pain, weakness, and dark tea-colored urine. Mild RM may be asymptomatic, but as the creatine kinase level rises beyond a certain threshold, severe complications such as electrolyte imbalance/disturbances, acute kidney injury (AKI), and disseminated intravascular coagulation may occur [3]. AKI is the most common and most severe complication of RM, and is reported to occur in 10–60% of patients with RM [4]. The occurrence of AKI can significantly worsen the prognosis of patients with RM, and the reported mortality rates of RM patients who develop AKI range from 7–80%[5]. Among RM patients in the intensive care unit, the mortality rate has been reported to be 59% when AKI was present and 22% when it was not [6].

In addition to conventional drug therapy, renal replacement therapy (RRT), including continuous or intermittent hemodialysis, hemofiltration, and hemodiafiltration, plays an important role in the treatment of RM-induced AKI. For patients with AKI, especially when associated with life-threatening complications such as hyperkalemia, hypercalcemia, hyperazotemia, anuria, or hyperhydration, RRT is indispensable [7]. RRT can remove uremic toxins and excess water, regulate the stability of the internal environment, and create a good environment for the recovery of renal and other organ functions. However, it is unclear whether RRT can prevent the occurrence of AKI in patients with RM. Although it has been reported that hemofiltration can effectively remove a certain amount of myoglobin (molecular weight, 17 kDa) from the blood of patients with RM [8, 9], most of these studies were case reports without control groups, and did not use the recovery of renal function and long-term prognosis as the study end-points. Upon searching the literature, we found no well-designed cohort study or randomized controlled trial probing the preventive effects of hemofiltration or hemodiafiltration on RM-induced AKI. Therefore, in this retrospective cohort study, we compared the effectiveness of continuous venovenous hemodiafiltration (CVVHDF) combined with standard medical therapy (SMT) versus SMT alone in preventing AKI in patients with RM. In addition, we conducted a health economic analysis of the two therapeutic schedules.

## Materials and methods

### Study design and patient selection

This retrospective single-center study involved patients who were diagnosed with RM and treated with or without CVVHDF in the Affiliated Hospital of Inner Mongolia Medical University, between January 2016 and December 2020. This study was approved by the ethics committee of the Affiliated Hospital of Inner Mongolia Medical University (no. 2,021,029). The inclusion criteria were determined according to the definition of RM [10, 11], and were as follows: (1) a clear etiological cause such as infection, trauma, poisoning, or heat sickness; (2) serum creatine kinase (CK) > 1000 IU/L or > 5 times the upper limit of normal; (3) presence of dark urine with or without limb swelling, weakness, fever, etc.; and (4) presence or absence of a significantly increased serum myoglobin level. The exclusion criteria were as follows: patients with newly diagnosed coronary heart disease, AKI, or chronic kidney disease (CKD). AKI was diagnosed using the criteria in the KDIGO Clinical Practice Guideline for Acute Kidney Injury [12]. According to this guideline, AKI is defined as any one of the following: (1) increase in serum creatinine by ≥ 0.3 mg/dL (≥ 26.5 µmol/L) within 48 h; (2) increase in serum creatinine to ≥ 1.5 times the baseline, which is known or presumed to have occurred within the prior 7 days; or (3) urine volume < 0.5 mL/kg/h for 6 h [12]. The severity of AKI was staged according to the following criteria [12]: stage 1, serum creatinine level 1.5–1.9 times the baseline or increased by ≥ 0.3 mg/dL (≥ 26.5 µmol/L), or urine output < 0.5 mL/kg/h for 6–12 h; stage 2, serum creatinine level 2.0–2.9 times the baseline or urine output < 0.5 mL/kg/h for ≥ 12 h; and stage 3, serum creatinine 3.0 times the baseline or increased to ≥ 4.0 mg/dL (≥ 353.6 µmol/L), or initiation of renal replacement therapy, or in patients aged < 18 years, a decrease in eGFR to < 35 mL/min per 1.73 m^2^, or urine output < 0.3 mL/kg/h for ≥ 24 h, or anuria for ≥ 12 h.

Among the patients selected according to the inclusion and exclusion criteria, the patients who were treated with CVVHDF combined with SMT formed the CVVHDF + SMT group. We, then, identified the patients who were treated using SMT only among the patients meeting the selection criteria as candidates for the SMT group. Finally, patients suitable for the SMT group were determined by matching the above candidates to the patients in the CVVHDF + SMT group in a 1:1 ratio according to age, gender, etiology, complications, and laboratory test indices on admission.

### CVVHDF and SMT

Details of the CVVHDF procedure are presented in Table 1. Before the initiation of CVVHDF, vascular access was secured by the venous insertion of a 12-French two-lumen hemodialysis catheter. The number of CVVHDF sessions was determined by the physician-in-charge based on the assessment of the patient’s condition. Each patient was hospitalized for approximately 1 week, and during this time, 2 or 3 CVVHDF sessions were conducted. The treatment was administered by well-trained nurses using a Baxter Prismaflex system and an M150 set, with an effective surface area of 1.5 m^2^ and a myoglobin-sieving coefficient of 0.70. Low-molecular-weight heparin was used for anticoagulation. A single treatment session lasted for 10–22 h. The dialysate flow rate was set at 1000 mL/h, and the total substitution fluid flow rate was 3000 mL/h. The substitution method consisted of both predilution and postdilution, with a median predilution substitution fluid flow rate of 2000 mL/h and a median postdilution substitution fluid flow rate of 1000 mL/h.

**Table 1:** Procedural characteristics of CVVHDF

Category	Product information
Device	
Equipment	Baxter Prismaflex system (Meyzieu Cedex, France)
Blood filter and extracorporeal circulation circuit	Baxter Prismaflex M150 set (Meyzieu Cedex, France)
**Vascular access**	
Type	Temporary catheter in right internal jugular vein or right femoral vein
Catheter	Able 12 Fr-16/20-cm double-lumen central venous catheter (Foshan, Guangdong)
**Anticoagulants**	
Drug	Low-molecular-weight heparin
First dose (IU)	3000 (2500–4750)
Additional dose (IU/h)	158 (100–200)
**Liquid piercing**	0.9% sodium chloride injection 2000 mL
**Blood flow rate (mL/min)**	160 (150–200)
**Dialysate or substitution fluid**	Qingshan Likang 4000 mL/bag (Chengdu, China)
**Dialysate flow rate (mL/h)**	1000
**Substitution method**	Combined predilution and postdilution
**Predilution fluid flow rate (ml/h)**	2000 (1000–2250)
**Postdilution fluid flow rate (ml/h)**	1000 (750–1500)
**Duration of single treatment (h)**	16 (10–22)
**Number of CVVHDF sessions**	
2	5 (56%)
3	4 (44%)
**Total number of treatments**	22
**Average number of treatments per patient**	2.44

Values are presented as median (25–75% interquartile range) or number (percentage)

CVVHDF, continuous venovenous hemodiafiltration

SMT mainly included full rehydration, alkalinization of the urine with sodium bicarbonate, and appropriate diuresis [13, 14]. In brief, the SMT consisted of the following measures. Fluid administration was initiated as soon as possible to maintain a urine output rate of 200–300 mL/h for at least the first 24 h. Intravenous sodium bicarbonate was administered to achieve a urine pH of 6.5–7.0, under arterial pH and serum bicarbonate monitoring, which should not exceed 7.5 and 30 mmol/L, respectively. A loop diuretic was used very prudently, and only under the condition of sufficient blood volume with limited urine output. Mannitol and antioxidant therapy were not used, and the underlying conditions and factors leading to RM were eliminated.

### Data collection

The demographic, clinical, and laboratory data of the patients were collected at admission and during the treatment process, and included the following parameters: serum myoglobin (Mb), serum CK, serum creatinine (Cr), blood urea nitrogen (BUN), serum potassium, and serum calcium. Renal survival data were collected for survival analysis; the terminal event for renal survival was defined as an increase in the serum Cr level over the upper limit of the normal range. Additionally, data on hospitalization expenses and length of stay were collected for the health economic analysis. Data collection was mainly accomplished using the Yidu cloud intelligent data system (Yidu Cloud Corporation, Beijing, China), which is an intelligent medical records system targeted for scientific research, and part of the data was obtained from the original medical records.

### Statistical analysis

We used IBM SPSS Statistics *v*20.0 and GraphPad Prism *v*8.0 for data analysis and graph generation. Categorical variables were expressed as numbers and percentages, and continuous variables were expressed as medians and interquartile ranges (25–75% quartiles), unless indicated otherwise. Variables were checked for normal distribution using the D’Agostino-Pearson omnibus normality test and the Shapiro-Wilk normality test. For comparisons, the chi-squared test, Mann-Whitney *U* test, Wilcoxon matched-pairs signed rank test, and two-sided paired *t* test were used, as appropriate. Renal survival in the two cohorts was analyzed using the Kaplan-Meier method and the log-rank test. All reported *P* values are two-sided unless indicated otherwise, and *P* < 0.05 was considered statistically significant.

## Results

### Cohort characterization

**Fig. 1:**
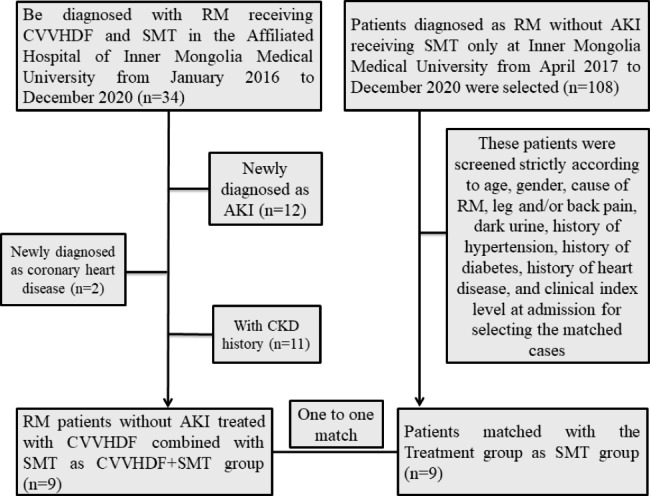
Flow chart of study participants. RM, rhabdomyolysis; CVVHDF, continuous venovenous hemodiafiltration; SMT, standard medical therapy; AKI, acute kidney injury; CKD, chronic kidney disease

Between April 2017 and December 2020, 34 patients were diagnosed with RM and treated with CVVHDF and SMT in the Affiliated Hospital of Inner Mongolia Medical University. After the exclusion of patients with newly diagnosed AKI (n = 12), newly diagnosed coronary heart disease (n = 2), and a history of CKD (n = 11), a total of 9 patients with RM and without AKI who had been treated with CVVHDF combined with SMT were included in the CVVHDF + SMT group. Among them, 5 cases received 2 CVVHDF treatment sessions and 4 cases received 3 CVVHDF treatment sessions. The CVVHDF treatment was administrated to patient every day or every other day (Table 1). We then selected 9 control subjects who were matched in a 1:1 ratio to the CVVHDF + SMT group patients in terms of age, gender, cause of RM, leg and/or back pain, dark urine, history of hypertension, history of diabetes, history of heart disease, and biochemical index levels at admission. All control patients had been diagnosed with RM without AKI and received SMT only at the Inner Mongolia Medical University during the same period as the SMT group. Figure 1 shows the flow chart for the selection of the patients enrolled in the two cohorts.

The demographic and clinical details of the subjects are summarized in Table 2. Of the 9 patients in the CVVHDF + SMT group, 8 (88.89%) patients were male. The age distribution of the patients in the CVVHDF + SMT group was as follows: 10–19 years, 2 (22.22%) patients; 20–29 years, 2 (22.22%) patients; 40–49 years, 1 (11.11%) patient; 60–69 years, 1 (11.11%) patient; and ≥ 70 years, 3 (33.33%) patients. The leading cause of RM was crush injury, followed by strenuous exercise and drugs. In the CVVHDF + SMT group, 1 patient had drug-induced RM caused by antidepressants (Doxepin hydrochloride tablet and lorazepam tablet). In the SMT group, 3 patients developed RM because of drugs, which included cold medicines (details unknown) in 1 patient, cold medicine plus traditional Chinese medicine (details unknown) in 1 patient, and an antidepressant and anti-schizophrenia drug (lorazepam tablet and haloperidol tablet) in 1 patient. Except for AKI, no other complications, such as compartment syndrome, coagulation disorder, and hypovolemic shock, were observed in any of our patients. Muscular soreness was present in 4 (44.44%) patients, and myohemoglobinuria was detected in 3 (33.33%) patients. The laboratory test results in the CVVHDF + SMT group were as follows: median serum Mb, 3000 ng/mL (2778.5–3000.0 ng/mL); median serum CK, 15,133 U/L (8881–123,845.5 U/L); median serum CK-MB, 639.8 U/L (149.4–1837.1 U/L); serum Cr, 96.889 ± 31.442 µmol/L; and BUN, 9.344 ± 5.110 mmol/L.

**Table 2:** Demographic and clinical characteristics at admission

Variable	CVVHDF + SMT group (n = 9)	SMT group (n = 9)	*P* value
**Sex**			0.730
Male	8 (88.89%)	7 (77.78%)	
Female	1 (11.11%)	2 (22.22%)	
**Age (years)**			0.546
10–19	2 (22.22%)	2 (22.22%)	
20–29	2 (22.22%)	1 (11.11%)	
30–39	0 (0.00%)	2 (22.22%)	
40–49	1 (11.11%)	1 (11.11%)	
50–59	0 (0.00%)	1 (11.11%)	
60–69	1 (11.11%)	0 (0.00%)	
≥ 70	3 (33.33%)	2 (22.22%)	
**Causes**			0.502
Crush injury	4 (44.44%)	3 (33.33%)	
Strenuous exercise	2 (22.22%)	3 (33.33%)	
Heat stroke	1 (22.22%)	0 (0.00%)	
Drugs	1 (11.11%)	3 (33.33%)	
CO poisoning	1 (11.11%)	0 (0.00%)	
**Clinical symptoms**			
Myohemoglobinuria			1.000
Yes	3 (33.33%)	3 (33.33%)	
No	6 (66.67%)	6 (66.67%)	
Muscular soreness			0.436
Yes	4 (44.44%)	7 (77.78%)	
No	5 (55.56%)	2 (22.22%)	
**Medical history**			
Hypertension history			1.000
Yes	1 (11.11%)	2 (22.22%)	
No	8 (88.89%)	7 (77.78%)	
Diabetes history			0.730
Yes	1 (11.11%)	0 (0.00%)	
No	8 (88.89%)	9 (100%)	
Heart disease history			0.730
Yes	0 (0.00%)	0 (0.00%)	
No	9 (100%)	9 (100%)	
**Laboratory indicators**			
Mb (ng/mL)	3000 (2778.5–3000.0)	2921 (805.7–3000.0)	0.796
CK (U/L)	15,133 (8881–123,845.5)	17,155 (8854.5–105,738.5)	0.931
CK-MB (U/L)	639.8 (149.4–1837.1)	601.3 (174.9–1859.3)	0.796
Cr (µmol/L)	96.889 ± 31.442	67.556 ± 9.002	0.139
BUN (mmol/L)	9.344 ± 5.110	5.967 ± 2.588	0.063
LDH (U/L)	875 (596.5–3773.5)	616 (550–2664.5)	0.222
ALT (U/L)	85 (55–356.3)	109.5 (82–268.2)	0.931
AST (U/L)	243 (169–1222.0)	300 (199–916.0)	0.931
WBC (10^9^/L)	15.898 ± 7.391	12.567 ± 5.227	0.147
HGB (g/L)	155.22 ± 16.581	151.22 ± 7.563	0.690
HCT (%)	45.6 (41.5–52.8)	43.5 (41.6–46.4)	0.796
CO_2_-cp (mmol/L)	20.433 ± 6.464	25.90 ± 4.348	0.153
Urine (pH)	5.5 (5.3–5.8)	6.5 (5.5–7.3)	0.063
d-Dimer (µg/mL)	1.77 (0.5–4.2)	0.6 (0.2–0.8)	0.136
CTnT (ng/mL)	0.09 (0.01–0.3)	0.003 (0.003–0.05)	0.063
Ca^2+^ (mmol/L)	2.108 ± 0.167	2.103 ± 0.169	0.345
K^+^ (mmol/L)	4.4 (4.2–4.5)	4.3 (2.8–4.5)	0.796
Na^+^ (mmol/L)	138.400 ± 4.410	138.330 ± 4.500	0.919
AG (mmol/L)	16.967 ± 11.507	11.100 ± 2.197	0.177

CVVHDF + SMT group, received continuous venovenous hemodiafiltration (CVVHDF) combined with standard medical therapy (SMT); SMT group, received SMT only. Values are presented as mean ± standard deviation, median (25–75% interquartile range), or number (percentage)

CO, carbon monoxide; Mb, myoglobin; CK, creatine kinase; CK-MB, creatine kinase-MB; Cr, creatinine; BUN, blood urea nitrogen; LDH, lactate dehydrogenase; ALT, alanine aminotransferase; AST, aspartate aminotransferase; WBC, white blood cell; HGB, hemoglobin; HCT, hematocrit; CO2-cp, carbon dioxide combining power; CTnT, cardiac troponin T; and AG, anion gap

Of the 9 patients in the SMT group, 7 (77.78%) patients were male. Their age distribution was as follows: 10–19 years, 2 (22.22%) patients; 20–29 years, 1 (11.11%) patient; 30–39 years, 2 (22.22%) patients; 40–49 years, 1 (11.11%) patient; 50–59 years, 1 (11.11%) patient; and ≥ 70 years, 2 (22.22%) patients. Muscular soreness was present in 7 (77.78%) patients, and myohemoglobinuria was detected in 3 (33.33%) patients. In the SMT group, laboratory tests returned the following results: median serum Mb, 2921 ng/mL (805.7–3000.0 ng/mL); median serum CK, 17,155 U/L (8854.5–105,738.5 U/L); median serum CK-MB, 601.3U/L (174.9–1859.3 U/L); mean serum Cr, 67.556 ± 9.002 µmol/L; and mean BUN, 5.967 ± 2.588 mmol/L. None of the above variables significantly differed between the two matched study groups.

### Comparisons between the CVVHDF + SMT and SMT groups

#### Biochemical parameters

**Fig. 2:**
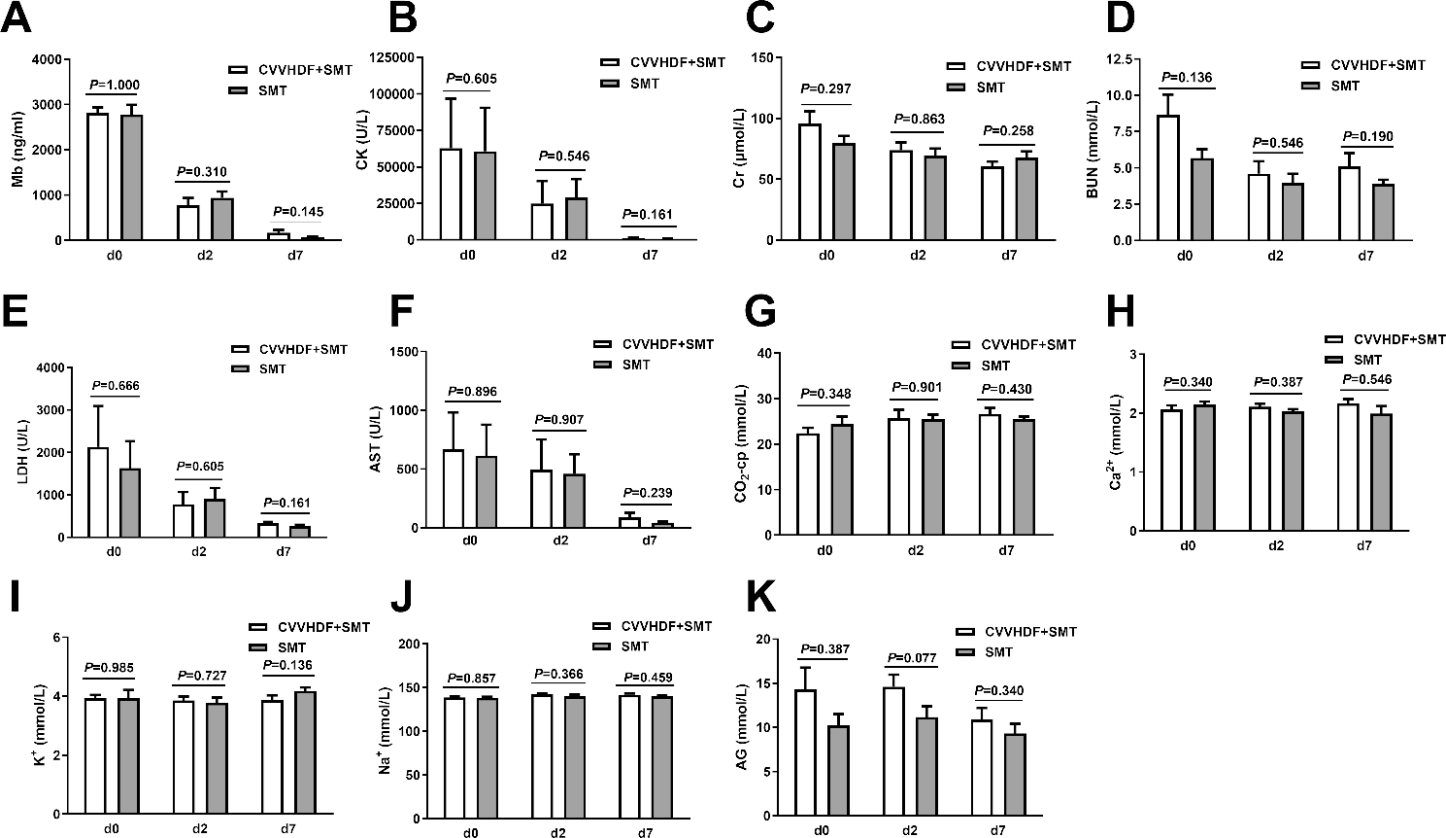
Comparison of biochemical indicators between the CVVHDF + SMT group and SMT group at different time points. CVVHDF + SMT group, received continuous venovenous hemodiafiltration (CVVHDF) combined with standard medical therapy (SMT); SMT group, received SMT only. D0, before the first CVVHDF treatment; d2 and d7, 24 h and 7 days after the first CVVHDF treatment. Some patients received 1 or 2 more treatment(s) after the first treatment. Mb, myoglobin; CK, creatine kinase; Cr, creatinine; BUN, blood urea nitrogen; LDH, lactate dehydrogenase; AST, aspartate aminotransferase; CO_2_-cp, carbon dioxide combining power; AG, anion gap

Biochemical indicators were compared between the CVVHDF + SMT and SMT groups before, and 24 h and 7 days after the first CVVHDF treatment (Fig. 2). We found no significant between-group differences in any biochemical indicator at any time point, including serum Mb, serum CK, serum Cr, BUN, serum lactate dehydrogenase (LDH), serum aspartate aminotransferase (AST), serum carbon dioxide combining power (CO_2_-cp), serum calcium, serum potassium, serum sodium, and serum anion gap (AG).

#### Renal survival

**Fig. 3:**
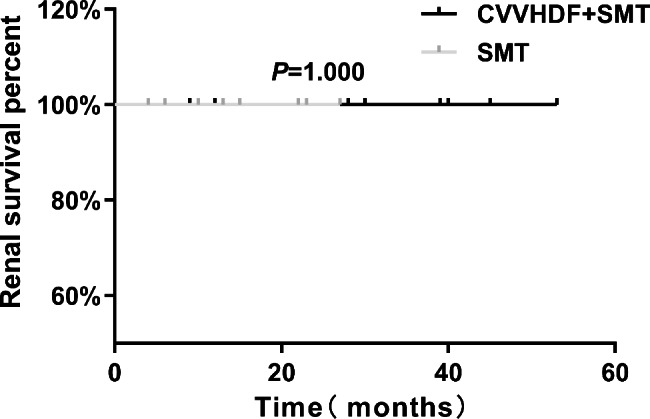
Comparison of renal survival rate after 53 months between the CVVHDF + SMT group and SMT group. CVVHDF + SMT group, received continuous venovenous hemodiafiltration combined with standard medical therapy (SMT); SMT group, received SMT only

All patients were followed up from hospital admission to January, 2021. The follow-up duration ranged from 53 to 4 months. One patient in the CVVHDF + SMT group died of acute myocardial infarction 12 months after hospital discharge. No other patient developed increased serum Cr (i.e., the terminal event) during follow-up. Renal survival analysis with the log-rank test showed no significant difference between the CVVHDF + SMT and SMT groups (*P* = 1.000; Fig. 3).

#### Health economics

**Fig. 4:**
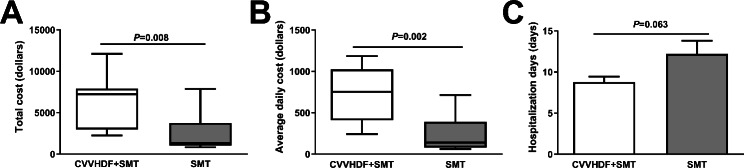
Comparison of health economic indexes between the CVVHDF + SMT group and SMT group. CVVHDF + SMT group, received continuous venovenous hemodiafiltration combined with standard medical therapy (SMT); SMT group, received SMT only

The indicators of health economics were compared between the CVVHDF + SMT and SMT groups (Fig. 4). The total cost (6446.70 ± 3184.45 vs. 2482.60 ± 2354.42 US dollars, *P* = 0.008) and average daily cost (744.87 ± 335.68 vs. 232.45 ± 228.37 US dollars, *P* = 0.002) during hospitalization were significantly higher in the CVVHDF + SMT group than in the SMT group. Moreover, the duration of hospitalization did not significantly differ between the CVVHDF + SMT and SMT groups (8.78 ± 1.99 vs. 12.22 ± 4.76 days, *P* = 0.063).

### Comparisons within the CVVHDF + SMT group

#### Biochemical indicators before and after the first CVVHDF treatment

**Fig. 5:**
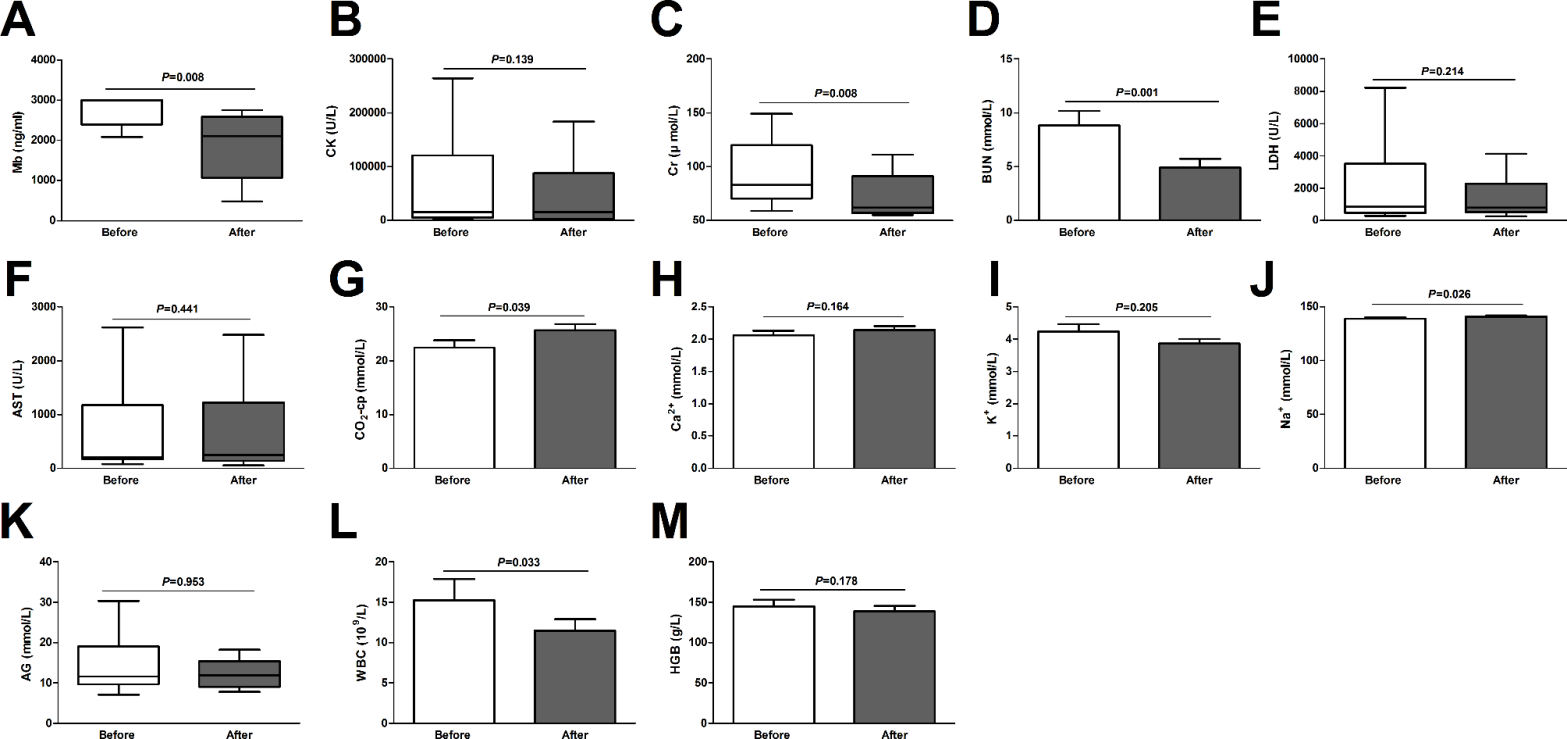
Comparison of biochemical indicators before and after the first continuous venovenous hemodiafiltration (CVVHDF) treatment in the CVVHDF + SMT group. CVVHDF + SMT group, received CVVHDF combined with standard medical therapy. Mb, myoglobin; CK, creatine kinase; Cr, creatinine; BUN, blood urea nitrogen; LDH, lactate dehydrogenase; AST, aspartate aminotransferase; CO2-cp, carbon dioxide combining power; AG, anion gap; WBC, white blood cell; HGB, hemoglobin

The comparison of biochemical indicators before and after the first CVVHDF treatment in the CVVHDF + SMT group is shown in Fig. 5. After the first CVVHDF treatment, the levels of serum Mb, serum Cr, BUN, serum sodium, and white blood cells (WBCs) significantly decreased, while the serum CO_2_-cp significantly increased compared to the pretreatment levels. Other indicators did not significantly change after the first CVVHDF session.

#### Biochemical indicators at different time points

**Fig. 6:**
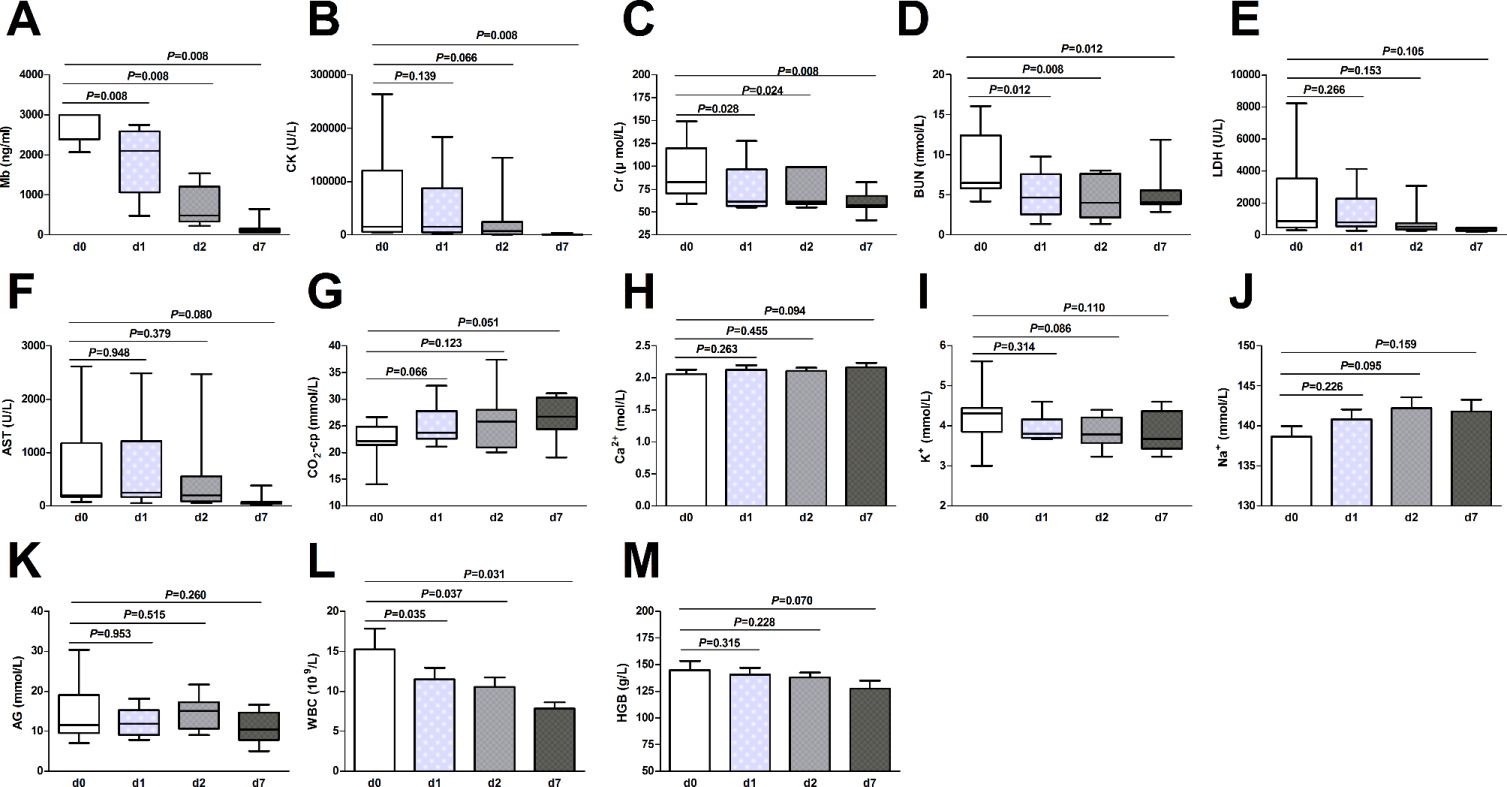
Comparison of biochemical indicators at different time points in the CVVHDF + SMT group. CVVHDF + SMT group, received continuous venovenous hemodiafiltration combined with standard medical therapy. D0, on admission; d1, d2 and d7, 1 day, 2 days, and 7 days after admission. Mb, myoglobin; CK, creatine kinase; Cr, creatinine; BUN, blood urea nitrogen; LDH, lactate dehydrogenase; AST, aspartate aminotransferase; CO_2_-cp, carbon dioxide combining power; AG, anion gap; WBC, white blood cell; HGB, hemoglobin

The comparison of the biochemical indicators on admission and at 1, 2, and 7 days after admission in the CVVHDF + SMT group is shown in Fig. 6. We found significant downward trends in the levels of serum Mb, serum CK, serum Cr, BUN, and WBCs; no trend was found in the other indicators.

## Discussion

To our knowledge, the present retrospective cohort study is the first to compare the effectiveness of CVVHDF combined with SMT vs. SMT alone in preventing the occurrence of AKI in patients with RM and to analyze the related health costs. Our findings demonstrate that although a single session of CVVHDF could facilitate myoglobin elimination, its addition to SMT did not significantly improve the serum Mb level or the levels of other biochemical indices or the long-term renal prognosis. However, the addition of CVVHDF to SMT did greatly increase the hospitalization costs.

While the therapeutic effects of RRT on RM-induced AKI have been extensively studied, its usefulness as a preventive measure is yet to be determined. Theoretically, currently available blood-purification techniques, such as high-flux hemodialysis, hemofiltration, hemodiafiltration, and plasmapheresis, can effectively eliminate myoglobin, which is a small protein molecule with a molecular weight of 17 kDa. Some studies have investigated the capacity of these blood-purification techniques to eliminate myoglobin. Sorrentino et al. measured the myoglobin clearance in 6 patients with RM-induced AKI, and found that high-flux hemodialysis effectively eliminated myoglobin, with a median myoglobin clearance of 90.5 mL/min (range, 52.4–126.3 mL/min) and a median myoglobin removal per treatment hour of 0.54 g (range, 0.15–2.21 g)[15]. Naka et al. reported the case of a 53-year-old woman with RM and AKI for whom hemofiltration resulted in a myoglobin clearance of 30.5–39.2 mL/min and removed 0.55–0.64 g myoglobin/treatment hour for 8 h of treatment [16]. In a case series of 6 patients with RM-induced AKI, the mean myoglobin clearance was 81 mL/min (range, 42–131 mL/min) after hemodiafiltration with a postdilutional fluid substitution rate of 2–3 L/h [17]. A control study with a two-stage crossover design also verified that hemodiafiltration could effectively clear myoglobin [18]. Although we did not find any study that confirmed that plasmapheresis eliminates myoglobin, we speculate that this is definitely the case because during plasmapheresis, plasma is non-selectively discarded and replaced with exogenous fresh plasma.

However, none of the above studies indicate whether these therapeutic measures can prevent the occurrence of RM-induced AKI because effective myoglobin clearance does not directly indicate a good prognosis [19]. Hence, cohort studies or randomized controlled trials with good control groups and renal survival data are required to directly investigate the ability of blood-purification techniques to prevent RM-induced AKI. We were unable to find any such studies via a literature search. We did find a systematic review that investigated the therapeutic effects of continuous renal replacement therapy (CRRT) for RM and RM-induced AKI [20]. This review included 4 studies from China, one of which contained patients without AKI and the other 3 studies contained patients with AKI. The review concluded that although CRRT may provide some benefits for RM patients, the poor methodological quality of the included studies and the lack of data on clinically important outcomes meant that there was insufficient evidence to discern any likely benefits of CRRT over conventional therapy for the prevention of RM-induced AKI [20]. A case report found that plasmapheresis did not prevent renal failure in a patient with RM-induced AKI [21]. We found no other study that explored the protective effects of plasmapheresis against RM-induced AKI. However, several studies have explored the therapeutic effects of plasmapheresis on RM-induced AKI and found that this treatment was effective [22, 23]. Nevertheless, as these studies were case reports or case analyses without a control group, Szpirt considered that the use of plasmapheresis was not justified for the treatment of RM and AKI [24].

CVVHDF is the most common CRRT technique, and combines hemofiltration and hemodialysis, so it could effectively clear both middle-molecular-weight urotoxins and micromolecular urotoxins, including myoglobin. Therefore, we conducted this retrospective cohort study to determine whether CVVHDF combined with the SMT vs. SMT only could prevent RM-induced AKI. Our study showed that although a single CVVHDF treatment facilitated myoglobin elimination, compared with SMT only, CVVHDF combined with SMT did not significantly improve the serum Mb levels or other biochemical indices or the long-term renal prognosis of patients with RM. We considered that the reason for the above was that although CVVHDF could eliminate some myoglobin effectively, the kidneys were still the main organs responsible for removing myoglobin under the condition of full hydration, urine alkalinization, and appropriate diuresis. On the basis of the results of our study and of other relevant studies, we consider that the available clinical evidence does not show any benefit of hemodiafiltration to prevent RM-induced AKI.

Interestingly, an animal experiment was performed to explore the direct renal protective effect of continuous venovenous hemofiltration (CVVH) in the early stage of RM [25]. In this study, the 2 hind legs of mongrel dogs were intramuscularly injected with 50% hypertonic glycerol to establish RM, and 2 h after the injection, CVVH was performed for 8 h. The study confirmed that at the cellular and molecular levels, CVVH treatment mitigated myoglobin-induced mitochondrial damage by inhibiting the mitochondrial apoptotic pathway and cell apoptosis; the treatment also delayed the occurrence of oliguria and protected the renal function during the early stage of RM development [25]. However, we consider that this single animal experiment cannot accurately represent the actual clinical condition due to the following reasons: First, in clinical practice, patients generally receive blood-purification treatments 1–2 days or even longer after the occurrence of RM, rather than being treated 2 h after the pathogenic onset like in the animal experiment; by the time blood-purification treatment is initiated, myoglobin has already caused some renal damage at the molecular level. Second, the CRRT regimen in most dialysis centers is 6–12 h daily or every other day for several days, so the observation period should be longer; however, in the animal experiment, observations were performed before and after a single treatment. Third, even with extracorporeal circulation therapy, the kidneys are still the main organs responsible for removing myoglobin.

In our study, we also conducted a health economic analysis, and found that although the total duration of hospitalization did not differ between the CVVHDF + SMT and SMT groups, both the total and daily hospitalization costs were significantly higher in the former than in the latter. Although few studies conduct health economic analyses, the cost of healthcare is a problem that cannot be ignored. CRRT, especially, is an expensive treatment requiring more medical insurance funds and patients’ financial resources, especially in less-developed areas.

The present study has certain limitations. First, this was a retrospective study with a small sample size. More large-scale prospective studies are required to explore this issue, including cohort studies and randomized controlled trials. Second, the ability of high-flux hemodialysis and plasmapheresis to prevent RM-induced AKI is also worth exploring.

## Conclusion

In summary, although a single CVVHDF treatment could facilitate myoglobin elimination, CVVHDF combined with SMT as compared to SMT only did not significantly improve the serum Mb levels or other biochemical indices like serum CK, Cr, BUN and LDH or the long-term renal prognosis. Although the total hospitalization duration did not differ between the two groups, CVVHDF combined with SMT obviously increased the total and daily hospitalization costs.

## Data Availability

The datasets used and/or analyzed during the current study are available from the corresponding author on reasonable request.

## Abbreviations

AG: Anion gap
AKI: Acute kidney injury
AST: Aspartate aminotransferase
BUN: Blood urea nitrogen
CK: Creatine kinase
CKD: Chronic kidney disease
Cr: Creatinine
CRRT: Continuous renal replacement therapy
CVVH: Continuous venovenous hemofiltration
CVVHDF: Continuous venovenous hemodiafiltration
CO: Carbon monoxide
CO_2_-cp: Carbon dioxide combining power
ICU: Intensive care unit
LDH: Lactate dehydrogenase
Mb: Myoglobin
RM: Rhabdomyolysis
RRT: Renal replacement therapy
SMT: Standard medical therapy
WBC: White blood cell
